# Antiviral response elicited against avian influenza virus infection following activation of toll-like receptor (TLR)7 signaling pathway is attributable to interleukin (IL)-1β production

**DOI:** 10.1186/s13104-018-3975-4

**Published:** 2018-12-04

**Authors:** Mohamed Sarjoon Abdul-Cader, Upasama De Silva Senapathi, Eva Nagy, Shayan Sharif, Mohamed Faizal Abdul-Careem

**Affiliations:** 1 Faculty of Veterinary Medicine, University of Calgary, Health Research Innovation Center 2C53, 3330 Hospital Drive NW, Calgary, AB T2N 4N1 Canada; 2 Department of Pathobiology, University of Guelph, Guelph, ON N1G 2W1 Canada

**Keywords:** Resiquimod, ssRNA, Macrophage, Avian influenza virus, Nitric oxide, Interleukin 1β

## Abstract

**Objective:**

Single stranded ribonucleic acid (ssRNA) binds to toll-like receptor (TLR)7 leading to recruitment of immune cells and production of pro-inflammatory cytokines, which has been shown in mammals. In chickens, ssRNA has been shown to elicit antiviral response against infectious bursal disease virus infection. The objectives of this study were to determine the pro-inflammatory mediators that are activated downstream of TLR7 signaling pathway in avian macrophages and their roles in antiviral response against avian influenza virus (AIV) infection.

**Results:**

In this study, first, we stimulated avian macrophages with the analog of ssRNA, resiquimod, and found that the ssRNA was capable of increasing nitric oxide (NO) and interleukin (IL-1β) production in avian macrophages. Second, we observed when the avian macrophages were stimulated with ssRNA, it elicits an antiviral response against AIV. Finally, we demonstrated that when we blocked the IL-1β response using IL-1 receptor antagonist (IL-1Ra) and the NO production using a selective inhibitor of inducible nitric oxide synthase (iNOS), *N*-([3-(aminomethyl)phenyl]methyl)ethanimidamide dihydrochloride (1400 W), the antiviral response against AIV is attributable to IL-1β production and not to the NO production. This study provides insights into the mechanisms of antiviral response mediated by ssRNA, particularly against AIV infection.

## Introduction

Macrophages are one of the major immune cell types involved in the innate immune system that recognize and eliminate various microbes. The microbial recognition by macrophages is mediated by the receptors expressed on macrophages referred to as pattern recognition receptors (PRRs) including various types of toll-like receptors (TLRs) [1–3]. In response to a virus infection, the TLRs recruit downstream adaptor molecules activating intracellular signaling cascades [4] with a consequence of upregulation of gene transcription for the production of pro-inflammatory molecules. The activated pro-inflammatory mediators includes antiviral cytokines such as interleukin (IL)-1β and inducible nitric oxide synthase (iNOS) [5–7]. The iNOS will facilitate production of a potent highly reactive antiviral free radical molecule, nitric oxide (NO), as a part of innate host defense against invading infectious agents [8, 9].

Of the many types of TLRs in birds, TLR7 is the only identified receptor that binds with viral single-stranded ribonucleic acid (ssRNA) or its synthetic analogs (i.e. resiquimod, imiquimod, gardiquimod and ioxoribine) [7, 10]. In chickens, ssRNA can induce antibacterial effects against *Salmonella Enteritidis* [11] and antiviral effects against infectious bursal disease virus infection [12, 13]. Recently, a study demonstrated that ssRNA upregulates mRNA expression of pro-inflammatory mediators including IL-1β and iNOS in chicken in vivo [14]. However, the antiviral response of TLR7 activation against avian influenza virus (AIV) infection is not known. AIV infections are prevalent globally causing severe diseases in birds and mammals including humans [15]. Therefore, our objectives of this study were to determine whether (1) activation of the TLR7 signaling pathway in avian macrophages produces pro-inflammatory molecules involved in antiviral activity and (2) these pro-inflammatory mediators are attributable to antiviral response against AIV infection in avian macrophages.

## Main text

### Materials and methods

#### Virus and TLR ligand

A low pathogenic AIV (LPAIV), A/Duck/Czechoslovakia/1956 (H4N6) with unknown number of passages was kindly provided by Dr. Eva Nagy (University of Guelph, Canada). Initially the virus was propagated in the embryonated chicken eggs at embryo day (ED)9–11 and the virus titer in the harvested allantoic fluid was determined by using standard plaque assay technique in Madin–Darby canine kidney epithelial (MDCK) cells. The ligand for TLR7, synthetic ssRNA, resiquimod (ssRNA), was purchased from Selleckchem (Houston, TX, USA).

#### Cells and cell culture

The Muquarrab Qureshi-North Carolina State University (MQ-NCSU) cell line [16], an avian macrophage cell line, was kindly gifted by Dr. Shayan Sharif (University of Guelph, Canada). This cell line was cultured in LM-HAHN medium as has been described previously [9]. Both Douglas Foster (DF)-1 [17] chicken fibroblast and MDCK cell lines, purchased from American Type Culture Collection (ATCC, Manassas VA, USA), were cultured in Dulbecco’s Modified Eagle’s Medium (DMEM) supplemented with penicillin (100 units/ml), streptomycin (100 µg/ml) and 10% fetal bovine serum (FBS), in an incubator at 40 °C and 5% CO_2_.

### Experimental design

#### Determining whether TLR7 ligand, ssRNA leads to increase in NO and IL-1β production in avian macrophages

MQ-NCSU cells were propagated in 12-well plates (1 × 10^6^ cells per well) for 24 h and subsequently stimulated with either Roswell Park Memorial Institute (RPMI) medium containing ssRNA (10 µg/ml) or only RPMI growth medium (control). The experiment was conducted including 3 replicates per group. The MQ-NCSU cell culture supernatants were collected at 24 h post-treatment. The cell culture supernatants were used to determine NO production from macrophages using Griess assay reagent system as described previously [9]. The experiment was performed two more times with similar results and the data were pooled.

For the quantification of IL-1β production following ssRNA treatment of avian macrophages, MQ-NCSU cells were cultured on coverslips in 12-well plates with 1 × 10^6^ cells per well. Subsequently after 24 h of culture, the cells were stimulated with either RPMI medium containing ssRNA (10 µg/ml) or only RPMI growth medium (control). The experiment was conducted including 3 replicates per group. Protein transport inhibitor cocktail (2 µl/ml) (cocktail of brefeldin A and monensin, eBioscience, San Diego, CA, USA) was added to culture medium following 6 h of incubation in order to prevent release of IL-1β to the extracellular space. After 24 h of stimulation, the cells were fixed with 4% paraformaldehyde, subsequently immunofluorescent staining for IL-1β was performed and analyzed the data as described previously [18]. The experiment was repeated two more times with similar results and the data were pooled.

#### Determining whether ssRNA-mediated antiviral response against H4N6 LPAIV is attributable to NO production

In this experiment, a selective inhibitor of iNOS, N-([3-(Aminomethyl)phenyl]methyl)ethanimidamide dihydrochloride (1400 W) (Sigma-Aldrich, St. Louis MO, USA) [19, 20] was used to block NO production. Initially, MQ-NCSU cells were cultured in 12-well plates for 24 h (1 × 10^6^ cells per well) and stimulated with RPMI growth medium containing either ssRNA (10 µg/ml), ssRNA (10 µg/ml) combined with 1400 W (100 µM), 1400 W (100 µM), or only RPMI growth medium as a control. The experiment was conducted including 3 replicates per group. Meanwhile, the MDCK cell was cultured in a separate 12-well plate for 24 h. The MQ-NCSU cell culture supernatants were collected at 24 h post-treatment and 250 µl of the collected cell culture supernatants were transferred on to MDCK cell monolayers before infecting with H4N6 LPAIV (50 PFUs/well). At 48 h post-infection, the plates were stained with 1% crystal violet and resulting plaques were counted. The remaining culture supernatants were used to determine NO production from macrophages using Griess assay reagent system as described previously [9]. The experiment was repeated two more times with similar results and the data were pooled.

#### Determining whether ssRNA-mediated antiviral response against H4N6 LPAIV replication is attributable to IL-1β production

MQ-NCSU cells were cultured in 12-well plates with 1 × 10^6^ cells per well for 24 h. The cells were incubated with RPMI medium containing ssRNA (10 µg/ml) or RPMI medium only (control). The experiment was conducted including 6 replicates per group. MQ-NCSU cell culture supernatants were collected at 24 h post-treatment and 250 µl of the cell culture supernatants were transferred to DF-1 cell monolayers. The receiving DF-1 cells were pre-incubated (30 min) with 1.2 µg/ml IL-1 receptor antagonist (IL-1Ra) (Kingfisher Biotech, Inc., CITY MN, USA). After 24 h of transferring MQ-NCSU culture supernatant, the DF-1 cells were infected with H4N6 LPAIV (0.1 MOI). Twenty-four hours post-infection, the infected DF-1 cell culture supernatants were collected from each well and titrated in MDCK cell monolayers in 10 fold serial dilution. The plates were stained with 1% crystal violet after 48 h and resulting plaques were counted. The experiment was repeated with similar results and the data were pooled.

#### Data analyses

For the purpose of identifying the differences between two groups, the Student’s *t* test (GraphPad Prism Software 5, La Jolla, CA, USA) was used. The one-way analysis of variance (ANOVA) followed by Bonferroni’s posttest for selected comparison was performed to identify the differences between groups when more than two groups were present in an experiment. The outlier test was conducted before being analyzed with each set of data using the Grubbs’ test (GraphPad software Inc., La Jolla, CA, USA). The differences between groups were considered significant at P ≤ 0.05.

### Results

#### Stimulation of avian macrophages with TLR7 ligand, ssRNA leads to increased production of NO and IL-1β

**Fig. 1:**
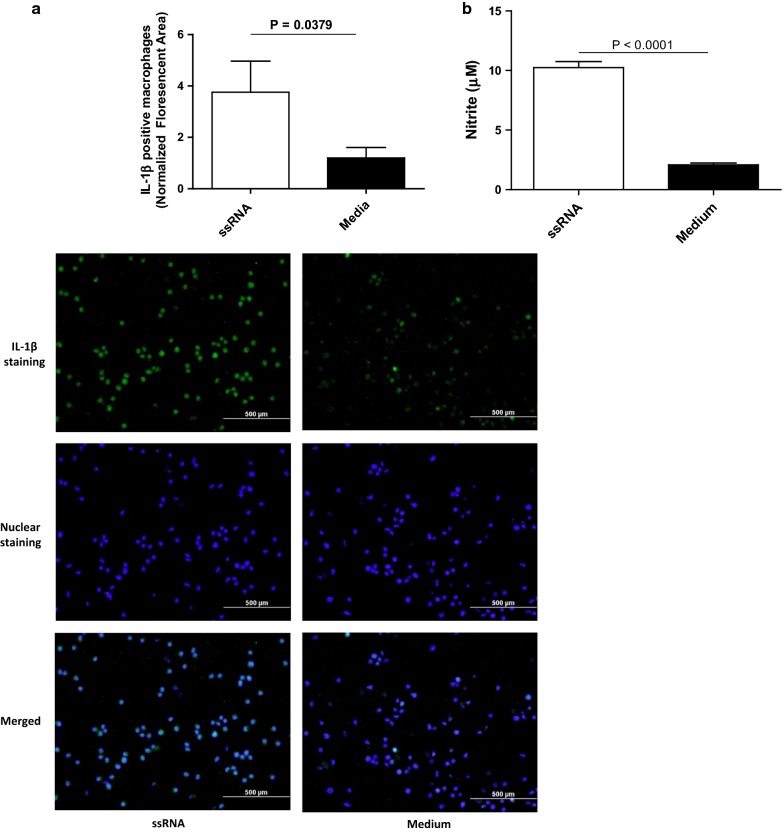
Stimulation of avian macrophages with TLR ligand, ssRNA increases the production of IL-1β and NO. **a** The MQ-NCSU cells were cultured on coverslips in 12-well plates with 1 × 10^6^ viable cells per well and protein transport inhibitor (2 µl/ml) was added to culture medium after 6 h. After 24 h of culture, the cells were stimulated with ssRNA (10 µg/ml), or RPMI growth medium as a control separately in triplicate. Following 24 h of treatment, immunofluorescent staining was performed for IL-1β expression after fixation with 4% paraformaldehyde. The representative figures in each group are shown as well the quantitative data of IL-1β expression. **b** MQ-NCSU cells were cultured in 12-well plates (1 × 10^6^ cells per well) for 24 h and stimulated with either ssRNA (10 µg/ml) or RPMI medium (control) in triplicate. The culture supernatants were collected 24 h post-treatment and NO assay was performed using Griess assay to quantify NO production. The experiment was repeated two more times with similar results and the data were pooled. Student’s *t*-test was used to identify differences between two groups and the differences were considered significant at P < 0.05. The bars represent mean ± SEM

In the current study, we evaluated avian macrophages as a source of NO and IL-1β productions in response to treatment with TLR7 ligand, ssRNA. We observed that ssRNA stimulation leads to higher production of IL-1β (Fig. 1a, P = 0.0379) and NO (Fig. 1b, P < 0.0001) in avian macrophages when compared to the controls.

#### Stimulation of avian macrophages with TLR7 ligand, ssRNA inhibits H4N6 LPAIV replication independent of NO production

**Fig. 2:**
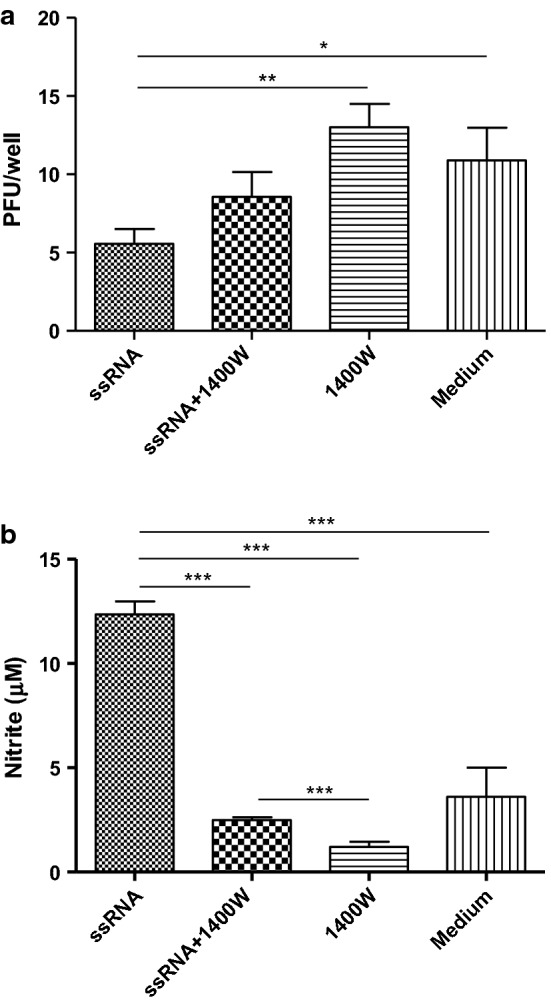
TLR7 ligand, ssRNA elicits antiviral response against H4N6 LPAIV in macrophages but not attributable to ssRNA-mediated NO production. **a** Avian macrophages, MQ-NCSU cells, were cultured in 12-well plates for 24 h and stimulated with either ssRNA (10 µg/ml), ssRNA (10 µg/ml) plus 1400 W (100 µM), 1400 W (100 µM) or growth medium (control) in triplicate. The cell culture supernatants were collected 24 h post-treatment, a portion was transferred onto MDCK cell mono layers grown in 12-well plates and infected with H4N6 LPAIV (50 PFU/well). **b** The NO assay was performed in remaining cell culture supernatants using Griess assay to quantify NO production. The experiment was repeated two more times with the same number of replicates with similar results and the data were pooled. The one-way ANOVA test followed by Bonferroni’s posttest for selected comparison was performed to identify group differences and the differences were considered significant at P < 0.05. The bars represent mean ± SEM

Then, we investigated whether stimulation of avian macrophages with ssRNA inhibits H4N6 LPAIV replication attributable to NO production. Cell culture supernatants derived from avian macrophages following stimulation with ssRNA for 24 h were able to elicit antiviral response against H4N6 LPAIV infection (Fig. 2a, P < 0.05) which correlated with a significant increase in NO production from macrophages (Fig. 2b, P < 0.05) when compared to the untreated controls. Furthermore, we observed that 1400 W mediated inhibition of ssRNA induced NO production in avian macrophages (Fig. 2b, P < 0.05) did not abrogate ssRNA-mediated antiviral response against H4N6 LPAIV (Fig. 2a, P > 0.05).

#### Antiviral response against H4N6 LPAIV replication elicited by TLR7 ligand, ssRNA in avian macrophages is attributable to IL-1β production

**Fig. 3:**
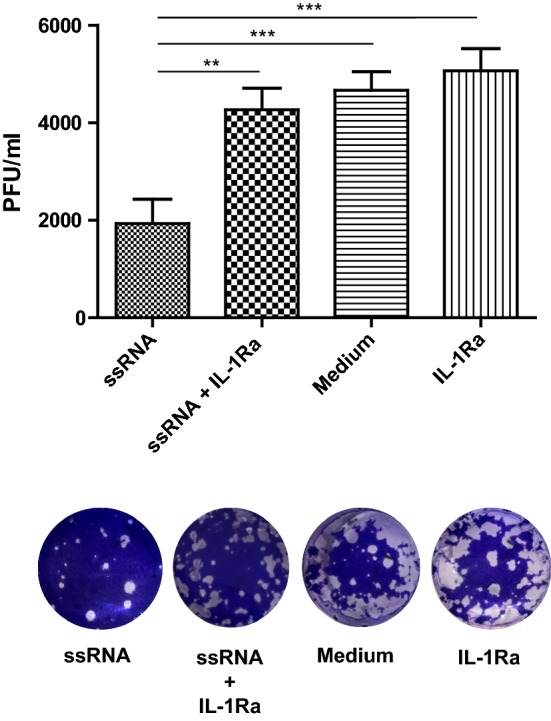
Antiviral activity of TLR7 ligand, ssRNA against H4N6 LPAIV replication is attributable to IL-1β production. Avian macrophages, MQ-NCSU cells, were cultured in 12-well plates for 24 h and stimulated with either ssRNA (10 µg/ml) or growth medium (control) including 6 replicates per treatment. The resultant MQ-NCSU cell culture supernatants were collected at 24 h post-treatment and transferred (250 µl) to DF-1 cell monolayers. Before transferring MQ-NCSU cell culture supernatants, 3 wells from ssRNA and control groups in DF-1 cells were incubated with 1.0 µg/ml of IL-1Ra for 30 min. Twenty-four hours later, the DF-1 cells were infected with H4N6 LPAIV (0.1 MOI). Twenty-four hours post-infection, the infected DF-1 cell culture supernatants were collected from each well and titrated in MDCK cell monolayers in tenfold serial dilution. The plates were stained with 1% crystal violet after 48 h and resulting plaques were counted. The experiment was repeated with the same number of replicates with similar results and the data were pooled. The representative picture of stained well in each group at 10^−1^ dilution are shown. The one-way ANOVA test followed by Bonferroni’s posttest for selected comparison was performed to identify group differences and the differences were considered significant at P < 0.05. The bars represent mean ± SEM

We then investigated to see whether stimulation of avian macrophages with TLR7 ligand, ssRNA inhibits H4N6 LPAIV replication in vitro attributable to IL-1β production. Here, we found that culture supernatants derived from avian macrophages following stimulation with ssRNA were able to inhibit H4N6 LPAIV replication compared to the controls (Fig. 3, P < 0.05) and blocking IL-1β signaling using IL-1Ra abrogated the antiviral response elicited against H4N6 LPAIV (Fig. 3, P < 0.05). Furthermore, we observed that blocking the IL-1β response following ssRNA stimulation did not significantly increase the H4N6 LPAIV replication when compared to the group that received only medium (Fig. 3, P > 0.05).

### Discussion

In this study, first, we found that the TLR7 ligand, ssRNA is capable of inducing avian macrophages leading to increased IL-1β and NO production. Second, the stimulation of avian macrophages with ssRNA is capable of eliciting antiviral response against H4N6 LPAIV replication. Third, the ssRNA-mediated antiviral response elicited against H4N6 LPAIV is attributable to IL-1 β production and not to the NO production.

Previously it has been shown that TLR ligands such as Cytosine-guanosine deoxynucleotides (CpG DNA), lipopolysaccharides (LPS) and lipoteichoic acid (LTA) were able to induce antiviral responses against AIV infection in chickens [9, 21]. Although, it has been shown that ssRNA can be antiviral against infectious bursal disease virus infection in chickens [12], there is a paucity of literature on the antiviral effect of TLR7 ligand, ssRNA against avian viral infections. Our study demonstrates that ssRNA was able to elicit antiviral responses against H4N6 LPAIV infection in avian macrophages.

As key immune cells involved in initiating innate antiviral response, avian macrophages are capable of producing highly reactive antiviral molecules such as NO [22]. In the current study, we observed that avian macrophages were capable of producing NO following stimulation with ssRNA. Previously it has been shown that NO originated from avian macrophages following stimulation with LPS and CpG DNA is inhibitory against LPAIV and ILTV infections [9, 23]. However, we did not find that ssRNA-mediated NO production is significantly attributable to antiviral response against H4N6 LPAIV. Although, this discrepancy in antiviral response mediated by NO originated from various TLR pathways is difficult to explain, it is possible that the difference may be connected to the amount of NO production downstream of these TLR signaling pathways. In the current study, the amount of NO produced from macrophages following ssRNA stimulation was < 15 µM. This NO production by avian macrophages is substantially lower when compared to NO production by other TLR ligands such as LPS [23] and CpG DNA [9] that ranged from 30 to > 50 µM respectively.

Avian macrophages also can produce a number of cytokines including IL-1β [24, 25]. In the current study, we observed that avian macrophages were capable of producing IL-1β following stimulation with ssRNA. Previously, it has been shown that the stimulation of avian macrophages with TLR21 ligand, CpG DNA, upregulates IL-1β mRNA expression [26]. Our current study also shows that IL-1β originated from avian macrophages in response to ssRNA treatment is attributable to antiviral response against H4N6 LPAIV infection. Although, IL-1β dependent antiviral response against avian viruses are not recorded previously, it has been reported that IL-1β inhibits the replication of West Nile virus [27], hepatitis B virus [28] and respiratory syncytial virus [29].

In conclusion, we have shown that the stimulation of avian macrophages with TLR7 ligand, synthetic ssRNA, is able to induce antiviral response against H4N6 LPAIV in vitro and this antiviral response is attributable to IL-1β production. Our results provide insights into the mechanisms of antiviral response mediated by ssRNA against H4N6 LPAIV infection in avian macrophages.

## Limitations

In spite of the novelty of the data providing insights into the mechanisms of ssRNA-mediated antiviral response, there are some potential limitations to our studies. First, although we studied the ssRNA-mediated antiviral response against H4N6 LPAIV and mechanistic aspect of this antiviral response, further studies are required to see whether ssRNA-mediated antiviral response is effective against highly pathogenic subtypes of AIV. Second, we do not know whether our observations were affected by the use of an LPAIV strain passaged unknown times since 1956. Therefore, further investigations should be done with a panel of more recent isolates of AIV. Finally, our in vitro findings should be substantiated by in vivo investigations.

## References

[1] Medzhitov R, Janeway CA (1997). Innate immunity: the virtues of a nonclonal system of recognition. Cell.

[2] Akira S (2003). Toll-like receptor signaling. J Biol Chem.

[3] Meylan E, Tschopp J, Karin M (2006). Intracellular pattern recognition receptors in the host response. Nature.

[4] Sasai M, Yamamoto M (2013). Pathogen recognition receptors: ligands and signaling pathways by Toll-like receptors. Int Rev Immunol.

[5] Haddadi S, Thapa S, Kameka AM, Hui J, Czub M, Nagy E, Muench G, Abdul-Careem MF (2015). Toll-like receptor 2 ligand, lipoteichoic acid is inhibitory against infectious laryngotracheitis virus infection in vitro and in vivo. Dev Comp Immunol.

[6] Uematsu S, Akira S (2007). Toll-like receptors and Type I interferons. J Biol Chem.

[7] Abdul-Cader MS, Amarasinghe A, Abdul-Careem MF (2016). Activation of toll-like receptor signaling pathways leading to nitric oxide-mediated antiviral responses. Arch Virol.

[8] MacMicking J, Xie QW, Nathan C (1997). Nitric oxide and macrophage function. Annu Rev Immunol.

[9] Abdul-Cader MS, Ahmed-Hassan H, Amarasinghe A, Nagy E, Sharif S, Abdul-Careem MF (2017). Toll-like receptor (TLR)21 signalling-mediated antiviral response against avian influenza virus infection correlates with macrophage recruitment and nitric oxide production. J Gen Virol.

[10] Philbin VJ, Iqbal M, Boyd Y, Goodchild MJ, Beal RK, Bumstead N, Young J, Smith AL (2005). Identification and characterization of a functional, alternatively spliced Toll-like receptor 7 (TLR7) and genomic disruption of TLR8 in chickens. Immunology.

[11] Swaggerty CL, He H, Genovese KJ, Duke SE, Kogut MH (2012). Loxoribine pretreatment reduces *Salmonella Enteritidis* organ invasion in 1-day-old chickens. Poult Sci.

[12] Annamalai A, Ramakrishnan S, Sachan S, Kumar BSA, Sharma BK, Kumar V, Palanivelu M, Varghese BP, Kumar A, Saravanan BC (2016). Prophylactic potential of resiquimod against very virulent infectious bursal disease virus (vvIBDV) challenge in the chicken. Vet Microbiol.

[13] Matoo JJ, Bashir K, Kumar A, Krishnaswamy N, Dey S, Chellappa MM, Ramakrishnan S (2018). Resiquimod enhances mucosal and systemic immunity against avian infectious bronchitis virus vaccine in the chicken. Microb Pathog.

[14] Annamalai A, Ramakrishnan S, Sachan S, Sharma BK, Anand Kumar BS, Kumar V, Badasara SK, Kumar A, Saravanan BC, Krishnaswamy N (2015). Administration of TLR7 agonist, resiquimod, in different types of chicken induces a mixed Th1 and Th2 response in the peripheral blood mononuclear cells. Res Vet Sci.

[15] Cardona CJ, Xing Z, Sandrock CE, Davis CE (2009). Avian influenza in birds and mammals. Comp Immunol Microbiol Infect Dis.

[16] Qureshi MA, Miller L, Lillehoj HS, Ficken MD (1990). Establishment and characterization of a chicken mononuclear cell line. Vet Immunol Immunopathol.

[17] Himly M, Foster DN, Bottoli I, Iacovoni JS, Vogt PK (1998). The DF-1 chicken fibroblast cell line: transformation induced by diverse oncogenes and cell death resulting from infection by avian leukosis viruses. Virology.

[18] Abdul-Cader MS, Amarasinghe A, Palomino-Tapia V, Ahmed-Hassan H, Bakhtawar K, Nagy E, Sharif S, Gomis S, Abdul-Careem MF (2018). In ovo CpG DNA delivery increases innate and adaptive immune cells in respiratory, gastrointestinal and immune systems post-hatch correlating with lower infectious laryngotracheitis virus infection. PLoS ONE.

[19] Jiang W, Pisetsky DS (2006). The role of IFN-alpha and nitric oxide in the release of HMGB1 by RAW 264.7 cells stimulated with polyinosinic–polycytidylic acid or lipopolysaccharide. J Immunol.

[20] Chiaradia LD, dos Santos R, Vitor CE, Vieira AA, Leal PC, Nunes RJ, Calixto JB, Yunes RA (2008). Synthesis and pharmacological activity of chalcones derived from 2,4,6-trimethoxyacetophenone in RAW 264.7 cells stimulated by LPS: quantitative structure-activity relationships. Bioorg Med Chem.

[21] Barjesteh N, Shojadoost B, Brisbin JT, Emam M, Hodgins DC, Nagy E, Sharif S (2015). Reduction of avian influenza virus shedding by administration of Toll-like receptor ligands to chickens. Vaccine.

[22] Setta A, Barrow PA, Kaiser P, Jones MA (2012). Immune dynamics following infection of avian macrophages and epithelial cells with typhoidal and non-typhoidal *Salmonella enterica* serovars; bacterial invasion and persistence, nitric oxide and oxygen production, differential host gene expression, NF-kappaB signalling and cell cytotoxicity. Vet Immunol Immunopathol.

[23] Haddadi S, Kim DS, Jasmine H, van der Meer F, Czub M, Abdul-Careem MF (2013). Induction of Toll-like receptor 4 signaling in avian macrophages inhibits infectious laryngotracheitis virus replication in a nitric oxide dependent way. Vet Immunol Immunopathol.

[24] Lavric M, Bencina D, Kothlow S, Kaspers B, Narat M (2007). Mycoplasma synoviae lipoprotein MSPB, the N-terminal part of VlhA haemagglutinin, induces secretion of nitric oxide, IL-6 and IL-1beta in chicken macrophages. Vet Microbiol.

[25] Alkie TN, Taha-Abdelaziz K, Barjesteh N, Bavananthasivam J, Hodgins DC, Sharif S (2017). Characterization of innate responses induced by PLGA encapsulated- and soluble TLR ligands in vitro and in vivo in chickens. PLoS ONE.

[26] Thapa S, Abdul-Cader MS, Murugananthan K, Nagy E, Sharif S, Czub M, Abdul-Careem MF (2015). In ovo delivery of CpG DNA reduces avian infectious laryngotracheitis virus induced mortality and morbidity. Viruses.

[27] Ramos HJ, Lanteri MC, Blahnik G, Negash A, Suthar MS, Brassil MM, Sodhi K, Treuting PM, Busch MP, Norris PJ (2012). IL-1beta signaling promotes CNS-intrinsic immune control of West Nile virus infection. PLoS Pathog.

[28] Isorce N, Testoni B, Locatelli M, Fresquet J, Rivoire M, Luangsay S, Zoulim F, Durantel D (2016). Antiviral activity of various interferons and pro-inflammatory cytokines in non-transformed cultured hepatocytes infected with hepatitis B virus. Antiviral Res.

[29] Bose S, Kar N, Maitra R, DiDonato JA, Banerjee AK (2003). Temporal activation of NF-kappaB regulates an interferon-independent innate antiviral response against cytoplasmic RNA viruses. Proc Natl Acad Sci USA.

